# A novel assay based on pre-equilibrium titration curves for the determination of enzyme inhibitor binding kinetics

**DOI:** 10.1007/s00249-021-01554-0

**Published:** 2021-06-22

**Authors:** Bernard Noppen, Anouk Vanbelle, Alan W. Stitt, Marc Vanhove

**Affiliations:** 1 Oxurion N.V, Gaston Geenslaan 1, 3001 Leuven, Belgium; 2 Present Address: Ordina Belgium, Blarenberglaan 3B, 2800 Mechelen, Belgium; 3 Centre for Experimental Medicine, Queen’s University Belfast, Northern Ireland, United Kingdom

**Keywords:** Enzyme inhibition assay, Pre-equilibrium inhibition curves, Tight-binding inhibitors

## Abstract

Selection of pharmacological agents based on potency measurements performed at equilibrium fail to incorporate the kinetic aspects of the drug–target interaction. Here we describe a method for screening or characterization of enzyme inhibitors that allows the concomitant determination of the equilibrium inhibition constant in unison with rates of complex formation and dissociation. The assay is distinct from conventional enzymatic assays and is based on the analysis of inhibition curves recorded prior to full equilibration of the system. The methodology is illustrated using bicyclic peptide inhibitors of the serine protease plasma kallikrein.

## Introduction

The intrinsic potency of a given drug is generally expressed on the basis of the affinity for its molecular target, and metrics such as IC_50_’s, K_D_’s or K_i_’s are broadly used to select and prioritize lead compounds. However, such parameters reflect an affinity measured at equilibrium and thus fail to describe the kinetic aspects of the drug–target interaction and the time-dependent changes in target engagement.

Macroscopically, the kinetics of drug–target interaction can be represented by the rate of complex formation (k_on_) and dissociation (k_off_). Similarly, the time a drug spends in contact with its biological target, referred to as residence time, reflects the inverse of the rate constant for drug–target unbinding (i.e., 1/k_off_) (Bernetti et al. 2017; Pan et al. 2013; Tonge 2018).

Recently, the kinetic aspects of drug–target interaction have received ever-growing attention from the drug discovery community following the observation that therapeutic efficacy can be significantly influenced by the kinetics of drug–target interaction and residence time (Bernetti et al. 2017, 2019; Pan et al. 2013; Tonge 2018; Di Trani et al. 2018; Shimizu et al. 2016; Zeilinger et al. 2017). Analytical methods aiming to provide accurate determination of the rate of drug–target complex formation and dissociation are thus expected to become part of the routine arsenal of tools for drug candidate screening and characterization.

Among potential molecular targets, enzymes, and in particular proteases, are regarded as highly attractive, the latter representing an estimated 5 to 10% of all pharmaceutical targets (Drag 2010). Here we report a method to study the interaction of enzyme inhibitors with their cognate target based on the analysis of pre-equilibrium inhibition curves. The method is distinct from conventional enzymatic assays, is applicable to highly potent molecules, and allows the determination of the kinetic parameters k_on_ and k_off_, together with the equilibrium inhibition constant K_i_, from a single experiment.

## Method

**Fig. 1:**
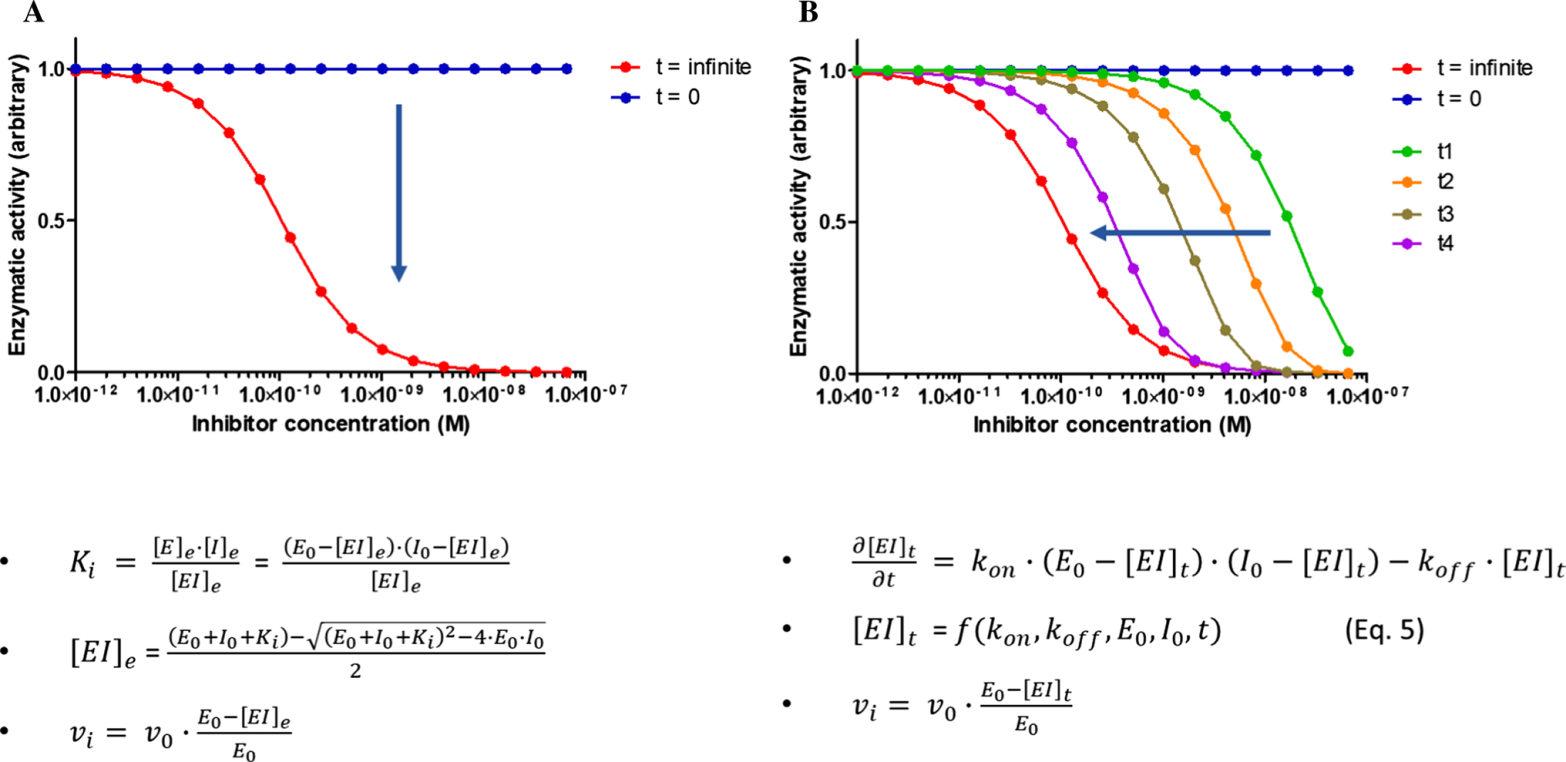
Illustration of equilibrium vs pre-equilibrium studies. **A** Data obtained at equilibrium are plotted as measured signal (e.g., enzymatic activity) vs. inhibitor concentration and analyzed using a model derived from the expression of the equilibrium constant. **B** Data obtained prior to equilibrium are plotted as measured signal vs. inhibitor concentration for different amounts of time and analyzed using a model derived from the differential equation describing the change in enzyme–inhibitor complex concentration over time. The dots in **A** and **B** are there for illustration purposes and were generated by mathematical simulation

The potency of reversible enzyme inhibitors is best described by the equilibrium inhibition constant K_i_. Poorly potent molecules can easily be studied experimentally under conditions where the total enzyme concentration E_0_ in the assay is smaller than the inhibition constant K_i_ (E_0_ <  < K_i_) and where the total concentration of inhibitor I_0_ used in the assay is larger than the total enzyme concentration (I_0_ >  > E_0_). Under these conditions, mathematically simplified binding models, obtained by assuming that the concentration of inhibitor at equilibrium I_e_ is equal to the total concentration of inhibitor (I_e_ = I_0_), can be used for data analysis and K_i_ determination. However, the development of highly potent molecules by the pharmaceutical industry often forces the experimental determination of K_i_ to be conducted under conditions where E_0_ ~ K_i_ or E_0_ > K_i_, and where I_0_ ~ E_0_. Under these conditions, analysis of experimental data, typically in the form of plots representing the residual enzymatic activity measured at equilibrium as a function of the inhibitor concentration (Fig. 1A), requires the use of non-simplified mathematical models derived from the theory of tight binding inhibitors, such as Eq. 1, were v_i_ and v_o_ represent the rate of substrate hydrolysis in the presence and absence of the inhibitor, E_0_ and I_0_ the total concentration of enzyme and inhibitor, and K_i,app_ the apparent inhibition constant (Lindhout et al. 1994; Teufel et al. 2018; Ulmer et al. 1995; Yiallouros et al. 1998). Incidentally, the way K_i,app_ is linked to the real inhibition constant depends on the inhibition mechanism; e.g., for competitive inhibition, K_i,app_ can be expressed as in Eq. 2, where [S] and K_m_ are the substrate concentration and the Michaelis–Menten constant of the enzyme for this particular substrate, respectively (Teufel et al. 2018; Masuda-Momma et al. 1993; Wilkes and Prescott 1985):

1 $$ {\text{v}}_{{{\text{i~}}}}  = {\text{v}}_{{\text{o}}} ~ \times ~\frac{{\text{1}}}{{{\text{E}}_{0} }} \times \left( {{\text{E}}_{0}  - \frac{{\left( {{\text{E}}_{{\text{0}}}  + {\text{I}}_{{\text{0}}}  + ~{\text{K}}_{{{\text{i,app}}}} } \right)~ - \sqrt {\left( {{\text{E}}_{{\text{0}}}  + {\text{I}}_{{\text{0}}}  + ~{\text{K}}_{{{\text{i,app}}}} } \right)^{{\text{2}}}  - {\text{4}}~ \times {\text{E}}_{{\text{0}}}  \times {\text{I}}_{{\text{0}}} } }}{{\text{2}}}} \right) $$

2 $$ {\text{K}}_{{{\text{i,app}}}}  = ~{\text{K}}_{{\text{i}}}  \cdot ~\left( {{\text{1}} + \frac{{\left[ {\text{S}} \right]}}{{{\text{K}}_{{\text{m}}} }}} \right) $$

Recently, Vanhove and Vanhove (2018) described mathematical equations which, adapted to the problem posed here, can be used to model the time-dependence of enzyme–inhibitor complex formation. The model is derived from the differential equation describing a 1:1 interaction between the enzyme E and the inhibitor I according to I + E ↔ IE (Eq. 3, with k_on_ and k_off_ the second order rate constant for complex formation and the first-order rate constant for complex dissociation, respectively):

3 $$ \frac{{{\text{d}}\left[ {{\text{EI}}} \right]_{{\text{t}}} }}{{{\text{dt}}}} = {\text{k}}_{{{\text{on}}}}  \cdot \left( {{\text{E}}_{0}  - \left[ {{\text{EI}}} \right]_{{\text{t}}} } \right) \cdot \left( {{\text{I}}_{0}  - \left[ {{\text{EI}}} \right]_{{\text{t}}} } \right) - {\text{k}}_{{{\text{off}}}}  \cdot \left[ {{\text{EI}}} \right]_{{\text{t}}} . $$

Vanhove and Vanhove provided an analytical solution to Eq. 4 which leads to the integrated equation expressing [EI]_t_, i.e., the concentration of enzyme–inhibitor complex at any time t, as a function of k_on_, k_off_, E_0_ and I_0_ (Eq. 5):

4 $$ \int \limits_{0}^{{\text{t}}} \frac{{{\text{d}}\left[ {{\text{EI}}} \right]_{{\text{t}}} }}{{{\text{k}}_{{{\text{on}}}}  \cdot \left( {{\text{E}}_{0}  - \left[ {{\text{EI}}} \right]_{{\text{t}}} } \right) \cdot \left( {{\text{I}}_{0}  - \left[ {{\text{EI}}} \right]_{{\text{t}}} } \right) - {\text{k}}_{{{\text{off}}}}  \cdot \left[ {{\text{EI}}} \right]_{{\text{t}}} }} = \int \limits_{0}^{{\text{t}}} {\text{dt}} $$

5 $$ \left[ {{\text{EI}}} \right]_{{\text{t}}}  = \frac{{{\text{a}} \cdot \left( {1 - {\text{c}}} \right) - {\text{b}} \cdot \left( {1 + {\text{c}}} \right)}}{{2 \cdot {\text{k}}_{{{\text{on}}}}  \cdot \left( {1 - {\text{c}}} \right)}} $$

with

$$ {\text{a}} = {\text{k}}_{{{\text{on}}}}  \cdot \left( {{\text{E}}_{0}  + {\text{I}}_{0} } \right) + {\text{k}}_{{{\text{off}}}} $$

$$ {\text{b}} = \sqrt {{\text{a}}^{2}  - 4 \cdot {\text{k}}_{{{\text{on}}}}^{2}  \cdot {\text{E}}_{0}  \cdot {\text{I}}_{0} } $$

$$ {\text{c}} = \left( {\frac{{{\text{a}} - {\text{b}}}}{{{\text{a}} + {\text{b}}}}} \right) \cdot {\text{e}}^{{ - {\text{b}} \cdot {\text{t}}}} $$

since

6 $$ {\text{v}}_{{\text{i}}}  = {\text{v}}_{0}  \cdot \frac{{{\text{E}}_{0}  - \left[ {{\text{EI}}} \right]_{{\text{t}}} }}{{{\text{E}}_{0} }} $$

combining Eq. (5) and (6) leads to Eq. 7, which can be used to analyze data representing the residual enzymatic activity measured as a function of the inhibitor concentration under pre-equilibrium conditions (Fig. 1B):

7 $$ {\text{v}}_{{\text{i}}}  = {\text{v}}_{0}  \cdot \frac{1}{{{\text{E}}_{0} }} \cdot \left( {{\text{E}}_{0}  - \frac{{{\text{a}} \cdot \left( {1 - {\text{c}}} \right) - {\text{b}} \cdot \left( {1 + {\text{c}}} \right)}}{{2 \cdot {\text{k}}_{{{\text{on}}}}  \cdot \left( {1 - {\text{c}}} \right)}}} \right). $$

Worth mentioning, Eq. 7 is obtained without any mathematical simplification and can thus be used to study highly potent molecules requiring experimental conditions where I_0_ ~ E_0_ (see discussion above). Another aspect of the method described here is that it does not require full equilibration of the system and it is, therefore, well suited for slowly equilibrating reactions, unlike conventional K_i_ measurements, where the time needed to reach the equilibrium may exceed the time during which the studied biomolecules are stable (Vanhove and Vanhove 2018).

**Fig. 2:**
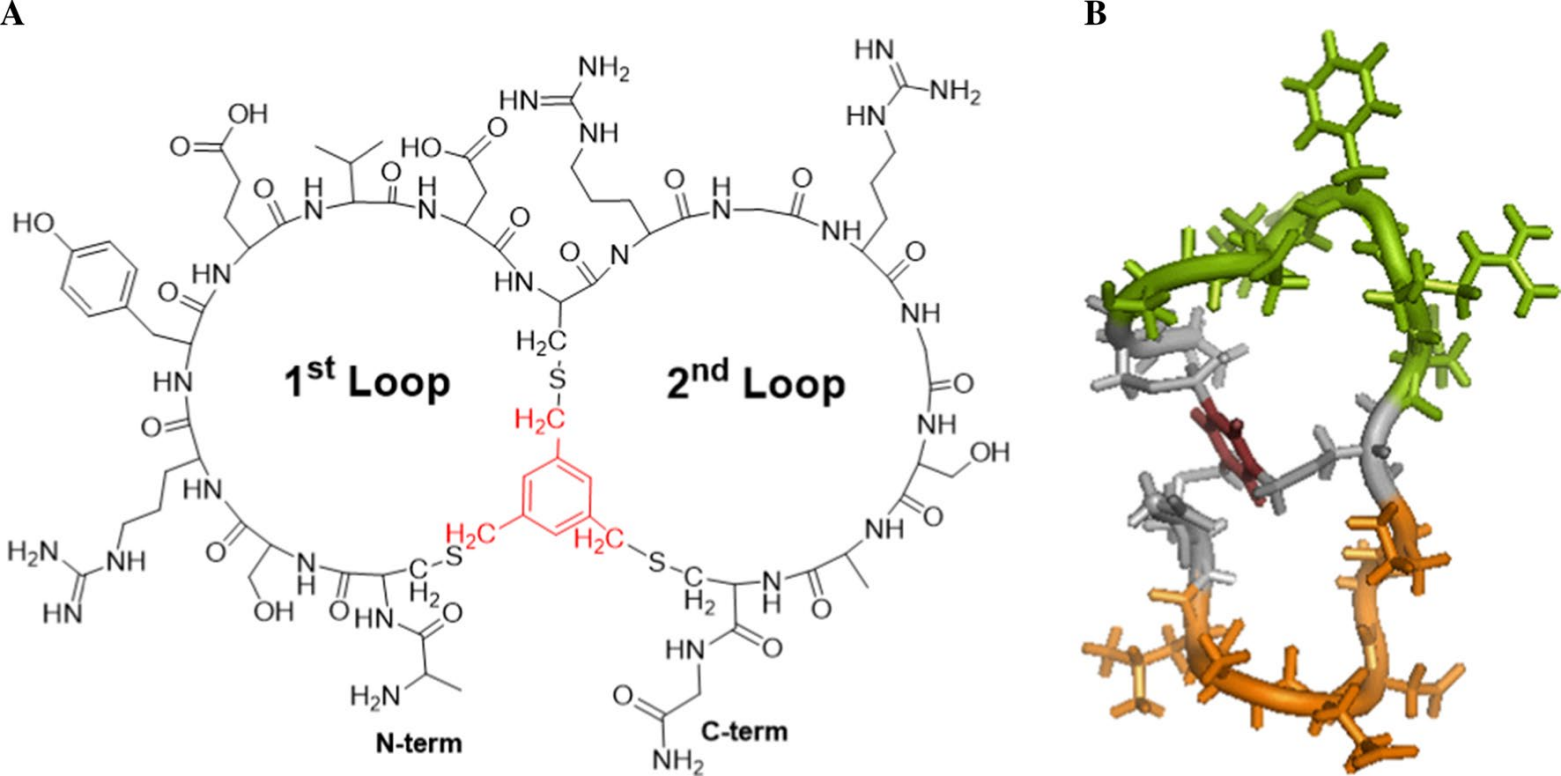
Representative structure **A** and 3D-fold **B** of 1,3,5-tris(bromomethyl)benzene (TBMB)-based bicyclic peptides. The structure shown in red (**A**) and dark brown (**B**) is the residual TBMB moiety following cyclization

We chose to characterize bicyclic peptide inhibitors of the serine protease plasma kallikrein (PKal) to evaluate the methodology described here in practice. Bicyclic peptides are constrained peptides consisting of a peptide sequence containing 3 cysteine residues which are covalently linked to a thiol-reactive molecular scaffold (Fig. 2) (Heinis et al. 2009; Rhodes and Dehua 2017). Bicyclic peptides are conformationally more constrained than their linear counterpart, and their preorganized, rigid structures confer high affinity binding, high specificity and superior stability (Chen et al. 2012). Furthermore, the use of structurally diverse cyclization reagents, such as 1,3,5-tris(bromomethyl)benzene (TBMB), 1,3,5-triacryoyl-1,3,5-triazinane (TATA), N,N′,N″-(benzene-1,3,5-triyl)-tris(2-bromoacetamide) (TBAB) or N,N′,N″-benzene-1,3,5-triyltrisprop-2-enamide (TAAB) that are able to impose different backbone conformations enables the identification of molecules which cover a wide range of chemical space (Chen et al. 2012).

**Fig. 3:**
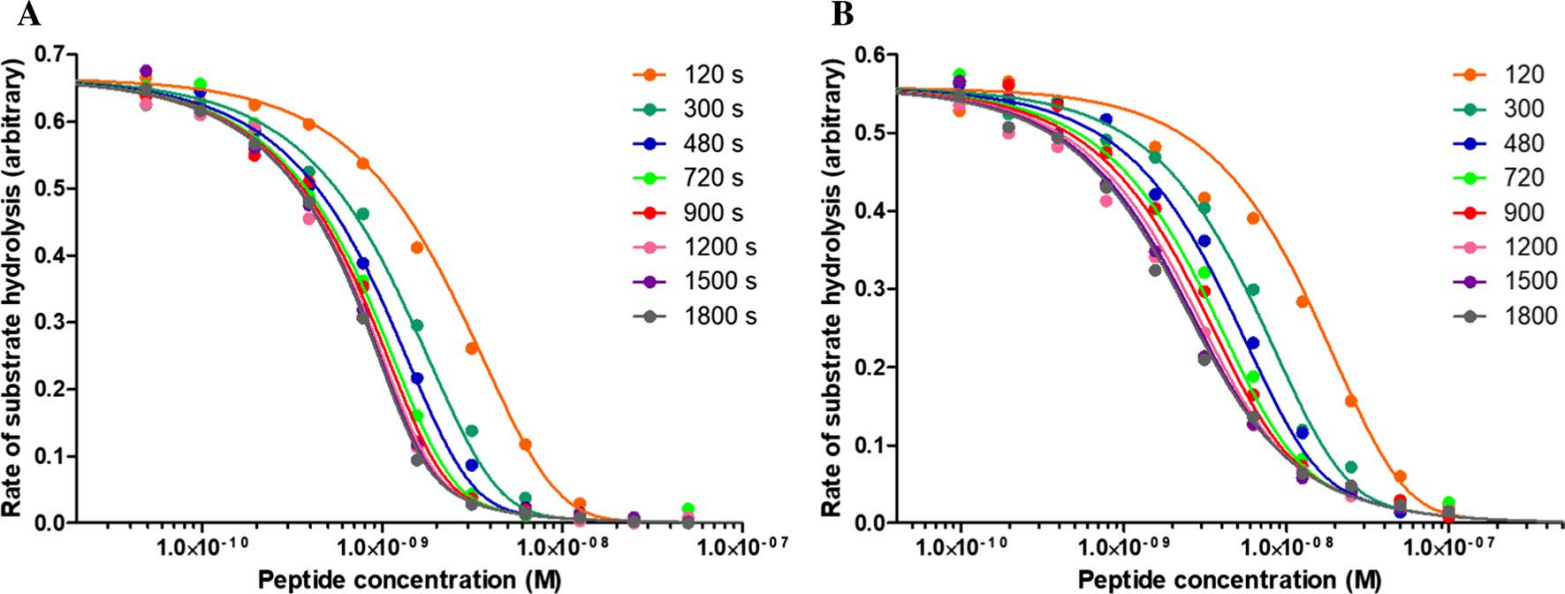
Inhibition of human plasma kallikrein by peptide 5c (**A**) and 1b (**B**) under pre-equilibrium conditions. Each dot corresponds to a single measurement of initial rate of substrate hydrolysis. The solid lines represent the best fit using Eq. 7. K_i_, k_on_ and k_off_ values obtained from these data are reported in Table 2

The TBMB-based bicyclic peptide inhibitors of PKal used in this study are listed in Table 1, some of which have been described previously (Teufel et al. 2018). Experiments were performed at 25 °C in 20 mM Tris–HCl, 150 mM NaCl, 1 mM EDTA, 0.1% PEG-6000, 0.1% Triton X-100, pH 7.5. Human PKal (Molecular Innovations, cat. HPKA, typical nominal concentration 1 nM for all peptides except 0.25 nM for peptide 4b) was pre-incubated in 96-well plates with different concentrations of the tested peptide (typically a serial twofold dilution plus a control with no peptide) in a reaction volume of 90 µL. At different timepoints, the fluorogenic substrate H-Pro-Phe-Arg-AMC (Bachem, cat. I-1295.0050, 10 µL, 20 µM final) was added to the relevant wells, and the increase in fluorescence at 480 nm (with excitation at 360 nm) was recorded using a Spectramax M2e plate reader (Molecular Devices). Initial rates of substrate hydrolysis were obtained by linear fit of the raw fluorescence vs. time traces. In addition, fluorescence was recorded ensuring that no more than 10% of the substrate were hydrolyzed, and for periods of time (typically 30–60 s) short enough to obtain linear traces. The data were plotted as the initial rate of substrate hydrolysis vs. peptide concentration for the different times of incubation prior to substrate addition (as shown in Fig. 3) and analyzed using Eq. 7.

**Table 1:** Plasma kallikrein bicyclic peptide inhibitors and their corresponding sequences

Peptide	Sequence, cyclized on C_i_, C_ii_, C_iii_ by TBMB
1b	Ac-C_i_NTWNPWC_ii_PWDAPLC_iii_A-Sar_3_-[D-Arg]_2_
2b	Ac-C_i_SWPARC_ii_LHQDLC_iii_
2c	Ac-C_i_SFPYRC_ii_LHQDLC_iii_
2d	Ac-C_i_SF[Aze]Y[HArg]C_ii_[Ala(ψCH_2_-NH)]HQDLC_iii_
4b	Ac-C_i_SF[Aze]Y[HArg]C_ii_VYYPDIC_iii_A-Sar_3_-[D-Arg]_2_
5a	Ac-C_i_SFPYRC_ii_LHQDLC_iii_
5b	Ac-C_i_SFPY[HArg]C_ii_LHQDLC_iii_
5c	Ac-C_i_SF[Aze]Y[HArg]C_ii_LHQDLC_iii_
5d	Ac-C_i_SF[Aze]Y[NMeArg]C_ii_LHQDLC_iii_

Peptides 1b, 2b, 2c, 2d and 4b were previously described (Teufel et al., 2018). Standard one letter code was used for natural amino acids. D-Arg: D enantiomer of arginine. Non-natural amino acids: Aze: azetidine-carboxylic acid, HArg: homoarginine, Sar: sarcosine, NMeArg: N-methylarginine. The first, second and third cysteine residues, which are cyclized by thioether formation with 1,3,5-tris(bromomethyl)benzene (TBMB), are designated as C_i_, C_ii_, C_iii_, respectively. All peptides are amidated C-terminally and contain an N-terminus capped by an acetyl group (referred to as “Ac”)

To compute unique values of k_on_ and k_off_, data sets such as those of Fig. 3 must be analyzed “globally”, i.e., considering all pre-equilibrium inhibition curves as a unique data set, as allowed by modern nonlinear regression analysis software’s. In this study, we used GraphPad Prism ver. 5.02 (GraphPad Software Inc., La Jolla, CA) applying equal weighting (i.e., performing minimization based on absolute distances squared), and we invite the reader to consult the software’s user’s manual for a detailed description of how global fitting of such data sets can be performed (https://cdn.graphpad.com/faq/2/file/Prism_v5_Regression_Guide.pdf). One point to highlight is that the parameters k_on_, k_off_ and E_0_ should be “shared”, to employ the terminology used in GraphPad Prism, i.e., that one unique value of these parameters is used to fit the data. By contrast, as far as the term v_0_ is concerned, we found that it can be either shared or allowed to take distinct values for the inhibition curves obtained at different timepoints to accommodate for a certain degree of experimental variability in initial rate measurements. As discussed previously for the analysis of data obtained under equilibrium, i.e., using Eq. 1 (see Teufel et al. 2018), the term E_0_ in Eq. 7 can be treated as a shared but adjustable parameter or, as here, fixed to its known value. The terms v_0_, k_on_ and k_off_, by contrast, always need to be treated as adjustable parameters. Finally, the knowledge of the kinetic parameters k_on_ and k_off_ enables calculation of the equilibrium inhibition constant K_i_ (K_i_ = k_off_/k_on_).

Strictly speaking, and similarly to measurements performed at equilibrium, all three parameters considered here, namely k_on_, k_off_ and K_i_, should be regarded as “apparent” (*app*) parameters, with their relationship to the actual parameters depending on the inhibition mechanism, e.g., for competitive inhibition, Eqs. 8–10 apply (Lindhout et al. 1994; Masuda-Momma et al. 1993; Yiallouros et al. 1998; Bakker et al. 1990):

8 $$ {\text{K}}_{{{\text{i,app}}}}  = ~\frac{{{\text{k}}_{{{\text{off,app}}}} }}{{{\text{k}}_{{{\text{on,app}}}} }} $$

9 $$ {\text{k}}_{{{\text{on,app}}}}  = \,\frac{{{\text{k}}_{{{\text{on}}}} }}{{{\text{1}} + \frac{{\left[ {\text{S}} \right]}}{{{\text{K}}_{{\text{m}}} }}}} $$

10 $$ {\text{k}}_{{{\text{off,app}}}}  = ~{\text{k}}_{{{\text{off}}}} $$

In the case of H-Pro-Phe-Arg-AMC, however, the K_m_ for human PKal is large (Teufel et al. 2018) and experiments were performed under conditions where [S] <  < K_m_. Therefore, the values for k_on_, k_off_ and K_i_ reported here can be considered as representing actual values.

## Results and discussion

**Fig. 4:**
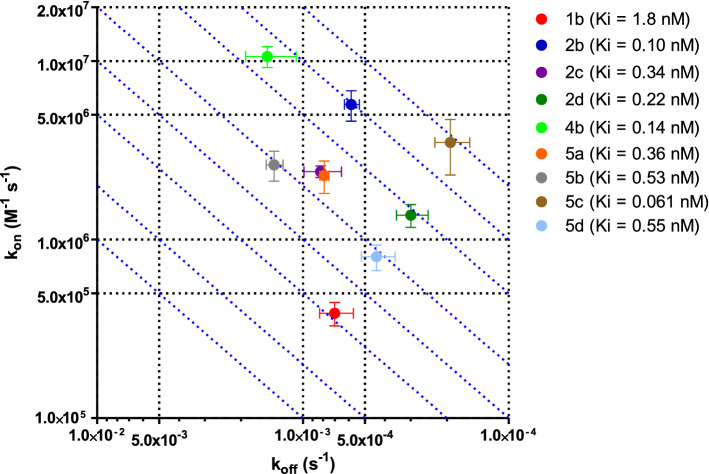
k_on_ vs. k_off_ plot for the inhibition of human plasma kallikrein by the indicated bicyclic peptides. Error bars represent standard deviations as from Table 2. Individual peptide potency (expressed as K_i_) is reported in the legend. The blue, dotted, diagonal lines graphically represent fixed K_i_ values defined from constant k_off_ vs. k_on_ ratios

Nine bicyclic peptides (Table 1) were tested for their ability to inhibit human PKal using the procedure described above. Representative data sets are shown in Fig. 3 for peptides 5c and 1b which are representative of higher and lower potency (lower and higher K_i_), respectively. Data fitting with Eq. 7 allowed the determination of the kinetic parameters k_on_ and k_off_ and, concomitantly, of the equilibrium inhibition constant K_i_ from a single experiment and a single set of data. The k_on_, k_off_ and K_i_ values for the tested peptides are reported in Table 2 and graphically in Fig. 4. The robustness of the method is demonstrated by the consistently low variability (average CV within 15–20%) of all measured parameters, whether k_on_, k_off_ or K_i_, obtained from independent measurements.

**Table 2:** Kinetic parameters k_on_ and k_off_ and equilibrium inhibition constant K_i_ for plasma kallikrein bicyclic peptide inhibitors. Values are reported as mean ± standard deviation of at least 3 independent measurements

Peptide	k_on_ (M^−1^ s^−1^)	k_off_ (s^−1^)	K_i_ (nM)	K_i_ (nM) from Teufel et al. 2018
1b	(3.86 ± 0.58) 10^5^	(7.0 ± 1.3) 10^–4^	1.8 ± 0.2	3.0 ± 0.3
2b	(5.7 ± 1.1) 10^6^	(5.82 ± 0.49) 10^–4^	0.10 ± 0.02	0.15 ± 0.18
2c	(2.40 ± 0.18) 10^6^	(8.2 ± 1.7) 10^–4^	0.34 ± 0.04	0.39 ± 0.17
2d	(1.37 ± 0.20) 10^6^	(2.99 ± 0.52) 10^–4^	0.22 ± 0.04	0.36 ± 0.07
4b	(1.06 ± 0.14) 10^7^	(1.49 ± 0.41) 10^–3^	0.14 ± 0.02	0.25 ± 0.20
5a	(2.28 ± 0.47) 10^6^	(7.88 ± 0.40) 10^–4^	0.36 ± 0.06	–
5b	(2.62 ± 0.50) 10^6^	(1.38 ± 0.13) 10^–3^	0.53 ± 0.07	–
5c	(3.5 ± 1.2) 10^6^	(1.92 ± 0.37) 10^–4^	0.061 ± 0.027	–
5d	(8.0 ± 1.3) 10^5^	(4.39 ± 0.82) 10^–4^	0.55 ± 0.06	–

**Fig. 5:**
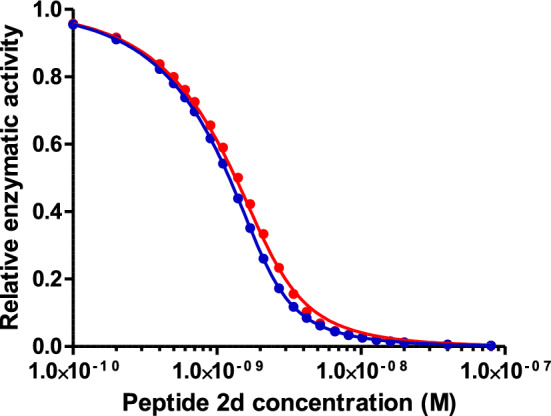
Red dots: inhibition titration curve for peptide 2d simulated using Eq. 7, assuming an enzyme concentration of 2 nM, k_on_ and k_off_ values as reported in Table 2, and an incubation time of 15 min. Blue dots: inhibition titration curve for peptide 2d at equilibrium, as predicted by either Eq. 1 or by Eq. 7 with t = ∞. Each set of data was separately analyzed with Eq. 1 (solid lines), leading to K_i_ values of 0.35 nM (t = 15 min) and 0.22 nM (t = ∞)

The K_i_ values reported here for peptides 1b, 2b, 2c, 2d and 4b are also in good agreement with those reported previously (Table 2; Teufel et al. 2018). K_i_ values reported by Teufel et al (2018) were obtained from conventional equilibrium measurements, i.e., by analyzing inhibition titration curves supposedly recorded at equilibrium using Eq. 1. Numerical simulations performed with Eq. 7 for, e.g., peptide 2d, however, show that an incubation time of 15 min as described by Teufel et al (2018), and under the conditions used by the authors (e.g., an enzyme concentration in the assay of 2 nM), is slightly less than the time needed to reach full equilibrium, and that this apparently very small deviation is sufficient to explain the difference in the Ki of peptide 2d reported in this study (0.22 ± 0.04 nM) compared to the one reported previously (0.36 ± 0.07 nM) (Fig. 5). A similar observation was made, e.g., for peptide 1b. This illustrates the advantage of the methodology proposed here, which eliminates the need to achieve complete equilibration of the studied system.

All tested peptides proved to be potent inhibitors of human PKal, with K_i_ values covering almost two orders of magnitude, ranging from single-digit nanomolar to double-digit picomolar values. Progression towards higher affinity (i.e., lower K_i_ values) resulted from both an increase in the rate of complex formation and a reduction in the rate of complex dissociation, both k_on_ and k_off_ evolving by ~ 1.0–1.5 order of magnitude. This is illustrated graphically by the fact that peptides with increasing affinities move towards the upper right corner of the k_on_ vs. k_off_ plot of Fig. 4.

Sagawa et al (2003) have reported that antibodies progressing along the affinity maturation pathway by somatic hypermutations exhibit an increase in affinity that is the result of a decrease in both the on-rate and the off-rate (with, however, a larger amplitude for the latter). This pattern is interpreted assuming that higher affinity antibodies have acquired a more rigid structure allowing the antibody–antigen interaction to shift from a “zipper” to a “lock-and-key” mechanism, with as a result a reduced entropic cost for complex formation. One can speculate, however, that because of their constraint structure, affinity improvement of bicyclic peptides such as achieved from repeated selection rounds of peptide-on-phage libraries is primarily enthalpy-driven.

Finally, it is worth noting that the value of k_on_ and k_off_ reported here for PKal bicyclic peptide inhibitors are in the same range as those typically observed for antibodies (see e.g., Steukers et al. 2006). This suggests that the interaction of bicyclic peptides with their enzyme targets closely resembles conventional protein–protein interactions which are influenced by contact surface area with the enzyme target and a diverse range of non-covalent interactions.

## Conclusion

With the recognition that the kinetics of drug–target interaction can influence therapeutic efficacy, the pharmaceutical industry is in need of robust assays which enable early and rapid screening of hits on the basis of both equilibrium and kinetic metrics. We propose a new methodology for the concomitant determination of the equilibrium inhibition constant K_i_ and of the association and dissociation rate constants k_on_ and k_off_ of enzyme inhibitors based on the analysis of inhibition curves recorded at different timepoints prior to equilibration of the system. The model used here is obtained without mathematical simplification and can thus be applied to highly potent (also referred to as tight binding) inhibitors. Another aspect of the method is that it does not require full equilibration of the system, making it particularly well suited for the investigation of slowly equilibrating systems and timeframes which may exceed the time during which the studied molecules are stable.
